# Different Conical Angle Connection of Implant and Abutment Behavior: A Static and Dynamic Load Test and Finite Element Analysis Study

**DOI:** 10.3390/ma16051988

**Published:** 2023-02-28

**Authors:** Győző Körtvélyessy, Árpád László Szabó, István Pelsőczi-Kovács, Tamás Tarjányi, Zsolt Tóth, Krisztina Kárpáti, Danica Matusovits, Botond Dávid Hangyási, Zoltán Baráth

**Affiliations:** 1 Department of Oral Biology and Experimental Dental Research, Faculty of Dentistry, University of Szeged, Tisza Lajos krt. 64-66, H-6720 Szeged, Hungary; 2 Department of Prosthodontics, Faculty of Dentistry, University of Szeged, Tisza Lajos krt. 64-66, H-6720 Szeged, Hungary; 3 Department of Medical Physics and Informatics, Albert Szent-Györgyi Medical School, Faculty of Science and Informatics, University of Szeged, Korányi fasor 9, H-6720 Szeged, Hungary; 4 Department of Orthodontics and Pediatric Dentistry, Faculty of Dentistry, University of Szeged, Tisza Lajos krt. 64-66, H-6720 Szeged, Hungary; 5 Department of Periodontology, Faculty of Dentistry, University of Szeged, Tisza Lajos krt. 64-66, H-6720 Szeged, Hungary

**Keywords:** titanium implant, dental implants, dynamic load, static load, FEA, conical angle, implant–abutment connection, screw loosening, dentistry, implantology

## Abstract

Dental implants are artificial dental roots anchoring prosthetic restorations to replace natural teeth. Dental implant systems may have different tapered conical connections. Our research focused on the mechanical examination of implant–superstructure connections. Thirty-five samples with 5 different cone angles (24°, 35°, 55°, 75°, and 90°) were tested for static and dynamic loads, carried out by a mechanical fatigue testing machine. Fixing screws were fixed with a torque of 35 Ncm before measurements. For static loading, samples were loaded with a force of 500 N in 20 s. For dynamic loading, the samples were loaded for 15,000 cycles with a force of 250 ± 150 N. In both cases, the compression resulting from load and reverse torque was examined. At the highest compression load of the static tests, a significant difference (*p* = 0.021) was found for each cone angle group. Following dynamic loading, significant differences (*p* < 0.001) for the reverse torques of the fixing screw were also shown. Static and dynamic results showed a similar trend: under the same loading conditions, changing the cone angle—which determines the relationship between the implant and the abutment—had led to significant differences in the loosening of the fixing screw. In conclusion, the greater the angle of the implant–superstructure connection, the smaller the screw loosening due to loading, which may have considerable effects on the long-term, safe operation of the dental prosthesis.

## 1. Introduction

Titanium (Ti) dental implants are used to replace the roots of missing teeth. Implant-based dentures have no harmful effects on neighboring teeth and provide an aesthetic prosthesis that is similar to natural teeth [1]. The load transmission mechanism on osseointegrated dental implants considerably differs from that of natural teeth: in the case of dental implants—that are fixed directly in the cortical and cancellous bone—there is no stress reduction (i.e., stress absorption) as with the case of periodontal ligaments in natural teeth, therefore, occlusal forces are transmitted directly to the surrounding bone [2]. As a result of reduced stress-bearing capacity, increased bone resorption rates and consequential peri-implant bone defects may develop more easily. An implant-supported dental restoration is a complex system, where the implant–abutment connection has a fundamental role in the long-term stability of the whole unit [3]. Due to occlusal forces, micro cracks and fractures may develop in the implant or in the connected elements [4]. Recurring mechanical forces may lead to reversible or irreversible changes to implant geometry, and, in addition, may lead to vertical and horizontal micro-movements between the implant and the abutment, which may result in screw loosening or screw fracture [3]. 

The forces acting on the dental prosthesis are distributed, they act on the superstructure, the implant (neck, wall thickness, body), the implant connection, and, subsequently, on the adjacent bone through different mechanisms and at different heights, depending on implant connection design and implant geometry [5]. Different implant connections may considerably affect the aforementioned force distribution. The transmission of forces and stress distribution are influenced by numerous factors, such as occlusion, the quantity and quality of the bone, implant body design, number and location of implants, implant inclination, osseointegration, abutment design, fit, and the micro-movements of the abutment, implant platform, thread design at the implant neck and implant–abutment connection, which may be external (EC) or internal (IC) [6,7]. One of the most common complications is the loosening and fracture of the fixing screw. Based on the literature data, screw fracture risk is higher in the case of EC, while the rate of long-term success is considerably higher in the case of IC [8,9]. A stable internal connection allows the implant body to be loaded, and by reducing the micro-movements, loosening and breaking may be avoided [10,11]. Biomechanically, the morse taper connection has proven to be the most load-resistant when measuring emerging stresses in the implant [12]. Morse connection consists of two interlocking cones in which the friction (cold welding) of the abutment and the body walls of the implant (self-closing connection) ensure that the load is distributed over a large surface [13]. Although morse taper connections may withstand large lateral forces, inhibiting the rotation of suprastructures is questionable, as the cross-section of the connection is circular, therefore, only friction inhibits rotation. Even with the use of octagonal indices, superstructure rotation may occur under heavy loads [13,14,15]. Lack of anti-rotation may result in subsequent loosening. Numerous studies have demonstrated a strong correlation between the free rotation of the abutment and the loosening of the implant–abutment interface [16]. Most implants used in recent years have an internal conical connection (ICC), although the angle of taper varies widely, and is often combined with an internal hexagonal or an octagonal connection [17]. From a clinical perspective, it is critical to understand how much of an effect these combinations have on implant stability. In many cases, different implant manufacturers use the same design of implant–abutment connection, as this simplifies the transition between different systems for clinicians in everyday practice [18]. Ideally, the reverse torque value does not change under the effect of load forces, nonetheless, screw loosening is a very common problem. We expect, from the connection, that the value of the reverse torque does not change due to the occlusal forces, but, in reality, the loosening of the screws is also a very common problem [19,20]. For this reason, there is no clear and ideal decision about the ideal taper angle for the implant–abutment connection. Additionally, there is also a limited understanding of whether a change in the value of the taper angle affects the loosening of the screws. During conical fitting, the flexibility of Ti results in a vertical displacement (compression), which may be followed by an irreversible dimensional change (plasticity) in the material of the implant neck [21,22]. It is important to clarify, for the practice, how the degree of conicity affects this material deformation. In addition, this deformation – due to the wedge effect – may cause the microcracking of the surrounding bone, as well as result in crestal bone loss and the loosening of the implant. Increasing the inner wall thickness of the implant body or reducing the diameter of the implant–abutment connection reduces the tension in the peri-implant bone. However, due to the internal geometry, this internal wall thickness may only be increased up to a certain limit [23,24]; the most commonly used narrow implants are in a diameter range of 3.3–3.8 mm, the implant wall width is less in case of smaller cone angle, which may increase the risk of irreversible dimensional change mentioned in the implant body [25].

The failure rate of implants due to static and dynamic loads is relatively high (32%) for implants with inadequate primary stability [26,27,28,29,30]. It is therefore critical to estimate the potential for failure in any given dental implant design. Experimental mechanical testing of dental implants provides useful data for engineers and physicists (involved in implant design) and clinicians [6,7,8]. In order to avoid failures in implant systems, it is important to have a detailed understanding of the mechanical behavior of the dental implants prior to their clinical application, which may be assessed via mechanical testing of the connection between the implant and the abutment. Parameters such as maximum allowable mechanical stress and reversible deformation, elastic limit, and fracture toughness are key indicators for determining the long-term durability of dental implant systems; thus, static and dynamic mechanical measurements should be performed [4,5,6,7,8]. Dental implant finite element analysis (FEA) is a computational method used to analyze the biomechanical behavior of dental implants. It is a powerful tool that allows dentists and dental engineers to simulate and analyze the stresses and strains that are exerted on the implant and the surrounding bone tissue. Dental implant FEA is used for a variety of purposes, such as optimizing implant design, predicting implant failure, and determining the optimal loading conditions for dental implants [31]. 

There are several publications on the impact of implant–abutment connections on implant success. Comparative studies of EC and IC were summarized in a systematic review and meta-analysis by Camps-Font et al. [32]. Similarly, Rodrigues et al. [33] published a systematic review and meta-analysis of randomized controlled trials (RCTs), where the results of ICC compared to internal non-conical connections (INCC); overall, no significant differences were found between the implant–abutment connections in terms of survival rate and biological complications. ICC showed greater preservation of peri-implant bone tissue and a lower probability of prosthetic complications than INCC and EC [32,33]. However, most studies have considered implants with different conical angle connections as one group, thus, the effects of different angles on clinical performance are poorly documented in the literature. In addition to peri-implant marginal bone loss and prosthetic complications, more data on implant survival and biological complications could be provided, if more future studies would focus on these angle deviations. Nonetheless, numerous studies indicate that implant diameter and length can considerably affect the stress distribution and occlusal load transfer at the bone–implant interfaces [34,35]; for example, based on clinical reports, the use of shorter implants presents with considerable disadvantages (i.e., lower success rate, survival), in contrast, there are considerable benefits of increasing implant length (while simultaneously keeping implant diameter constant) in enhancing bone–implant contact area and primary stability, up to a cut-off point of around 12–15 mm [36]. 

Our current study had the following research questions: (*i)* what is the degree of conicity at which screw loosening and deformation may be expected at the implant neck due to external forces? *(ii)* what are the differences in the mechanical stability of dental implants with tapered connections generated by varying the internal taper angle values?

## 2. Materials and Methods

### 2.1. Instruments 

The static and dynamic load tests were performed with a fatigue machine (Instron ElectroPuls E3000, Norwood, MA, USA). Grade 4 titanium implants with a 3.4 mm diameter were selected for this study, with the following cone angles: 24°, 35°, 55°, 75°, and 90°. A total of 35 implant samples were used for both static and dynamic load tests. To measure extension torque, a BMS MS150 electric torque screwdriver (BMS Torque Solutions, Limerick, Ireland) was used.

### 2.2. Static Load Test Protocol

**Figure 1:**
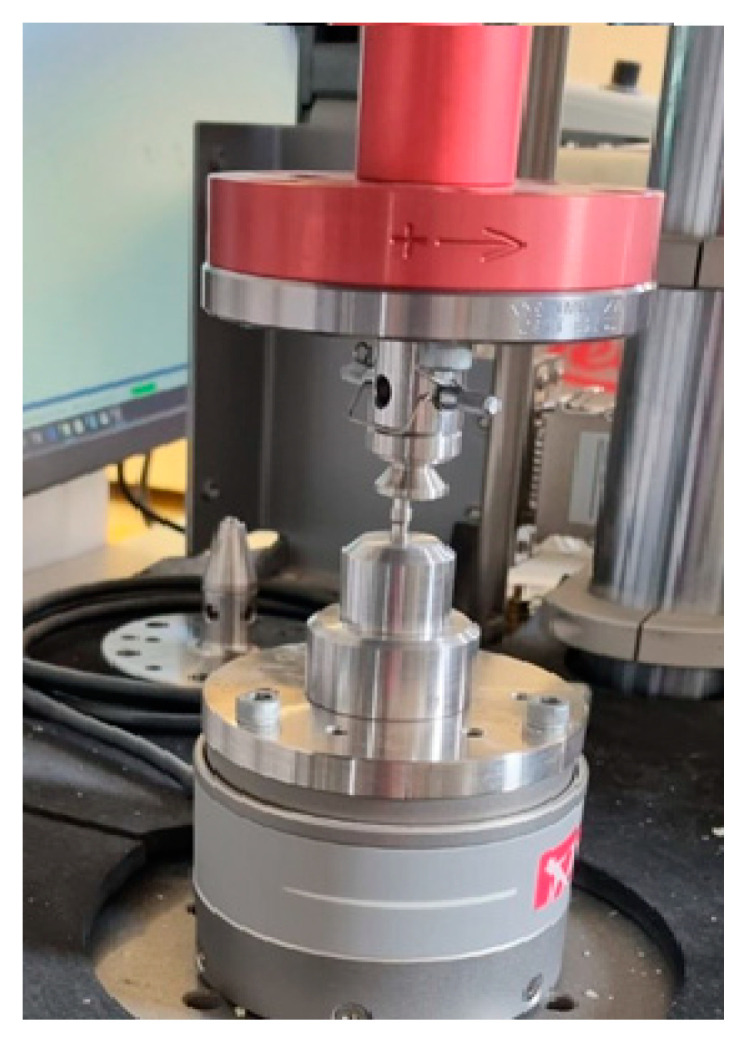
Fatigue machine used during the experiments and the setup of the static load test. The loading head was perpendicular to the top surface of the implant head.

The aim of our static load test was to investigate how the implant–abutment connection of implants with an identical design but different conical angles behave under load. The implant and the abutment head were tightened with the fixing screw, with a torque of 35 Ncm. The specimens were then placed in a special box, fabricated for this experiment, that held them under the load head during loading. The samples were then pressed by the machine, the force being perpendicular to the surface of the implant abutment, as seen in Figure 1. The compression value reading (in N) was obtained by the machine from the position of the loading head. During the load test, the load was gradually increased to 500 N over 20 s, and after reaching this peak force, the load was decreased back to 0 N over another 20 s. After each load test, the extension torque of the fixing screw was measured by an electric torque screwdriver. From the obtained load curves, resilience and energy dissipation were calculated from the area under the curve (AUC), with a numerical method.

### 2.3. Dynamic Load Test Protocol

After the static load test, the same samples were used for further fatigue tests. In the dynamic load test, occlusal forces were modeled, therefore, the tests were performed in similar force ranges that may affect the implants in the oral cavity. On each implant, the first step of our measurement was the tightening of the fixing screw, which holds together the implant abutment and the implant, with 35 Ncm torque [37]. Following this, the implants were put under loading. In the first phase, the fatigue machine loaded with 0.25 kN (equivalent to 25 kg) force over 10 s on the abutment. The dynamic load test started following the first phase; during the test, a 0.15 kN (equivalent to 15 kg) amplitude sine wave was applied with a 10 Hz frequency. This resulted in a dynamically changing force between 0.1 kN and 0.4 kN over time. The fatigue test lasted 15,000 cycles. After the fatigue test was performed, the force was released over another 10 s. Following this process, each implant and abutment were unscrewed and the torque was measured.

### 2.4. Finite Element Analysis (FEA)

**Figure 2:**
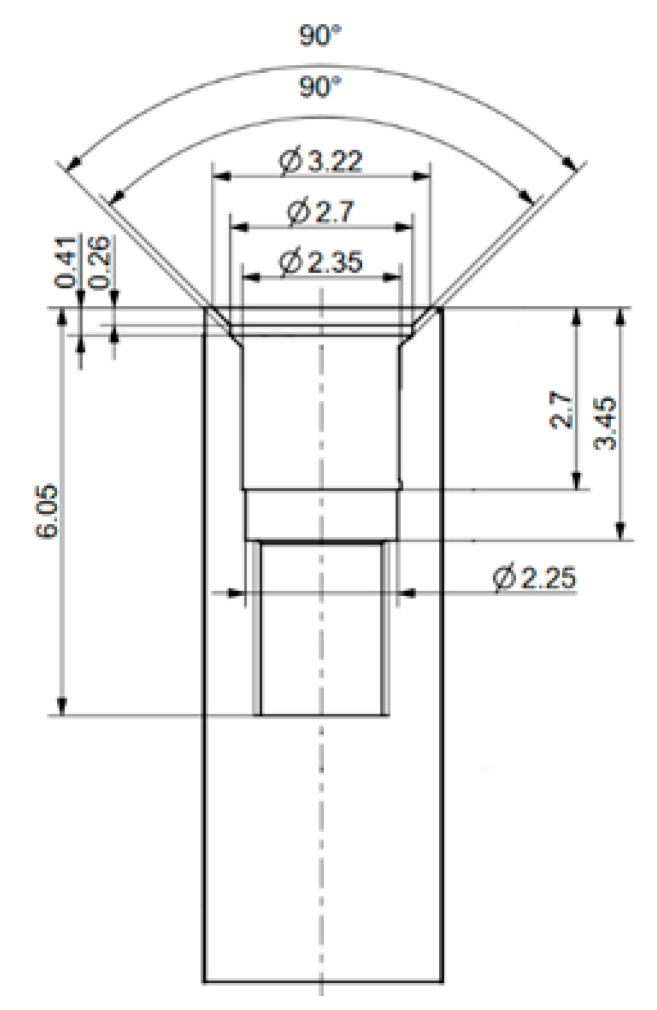
The schematic model of the 90° implant with dimensions (in mm).

**Figure 3:**
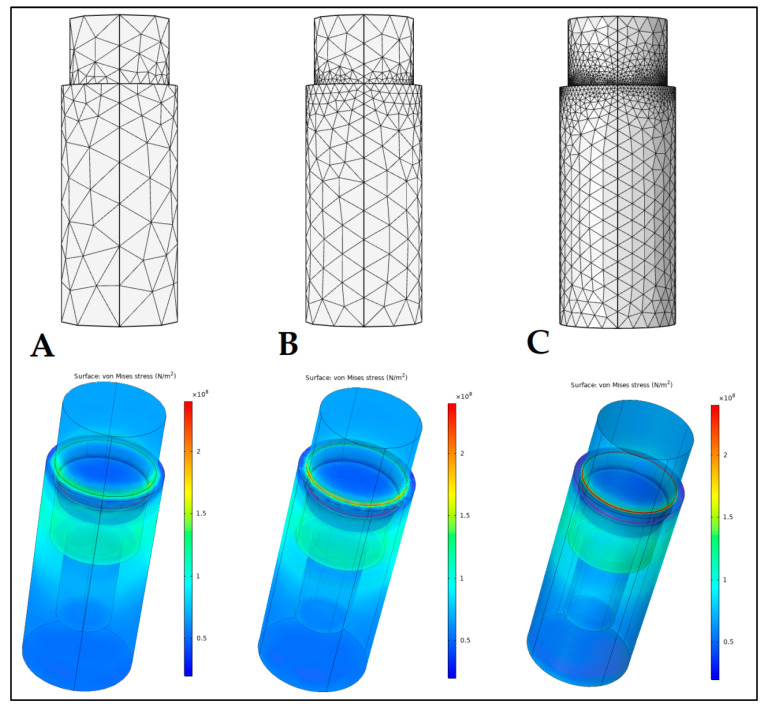
Different meshing options (**A**: coarse; **B**: normal; **C**: finer) and the resulting stress distribution figures.

A preliminary FEA was performed to examine the mechanical stress occurring in the implant, in the case of different cone angles. For the purposes of FEA, the COMSOL Multiphysics 5.5 software (COMSOL Inc., Burlington, MA, USA) was utilized. For the study, the 24° and 90° cone angle implant and abutment models were modeled, based on the manufactured samples that were presented in this study (Figure 2). In our analysis, the implant and the abutment model were interpreted as one body with a perfect tight fit. The mesh was created and adjusted by the built-in physics-controlled sequence of the COMSOL software, while the element size was set to normal in the settings [38]. Mesh-stress convergence was tested by adjusting the different COMSOL meshing options (coarse: number of elements: 2520, triangles: 1282; normal: number of elements: 10,589, triangles: 3692; finer: number of elements: 91,772, triangles: 17,310) (Figure 3). Overall, we may observe that the final stress distributions resulting from the different meshing size options did not diverge drastically, i.e., they all showed similar results (Figure 3). Subsequently, the normal meshing option was selected for further analyses: the number of elements (tetrahedra) was 10589, with a mesh volume of 63.06 mm^3^, average element quality of 0.5975, and an element volume ratio of 1.29 × 10^−4^, respectively. 

**Figure 4:**
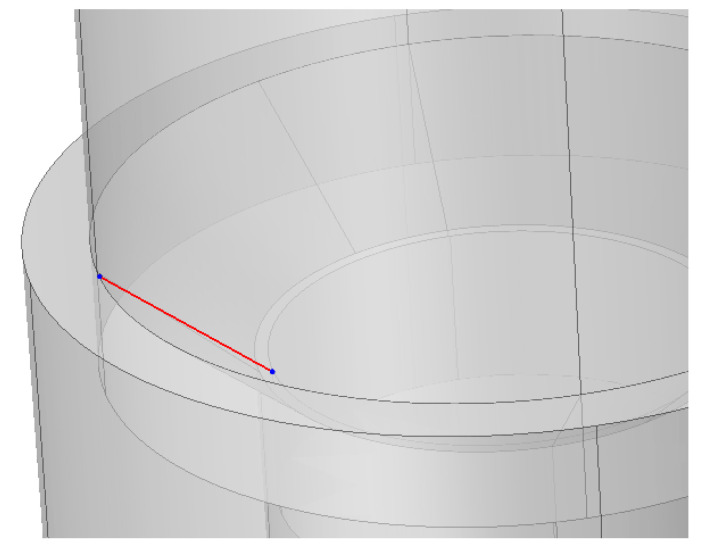
The line defined on the conical interface in the case of a 90° connection to assess mechanical stress.

**Figure 5:**
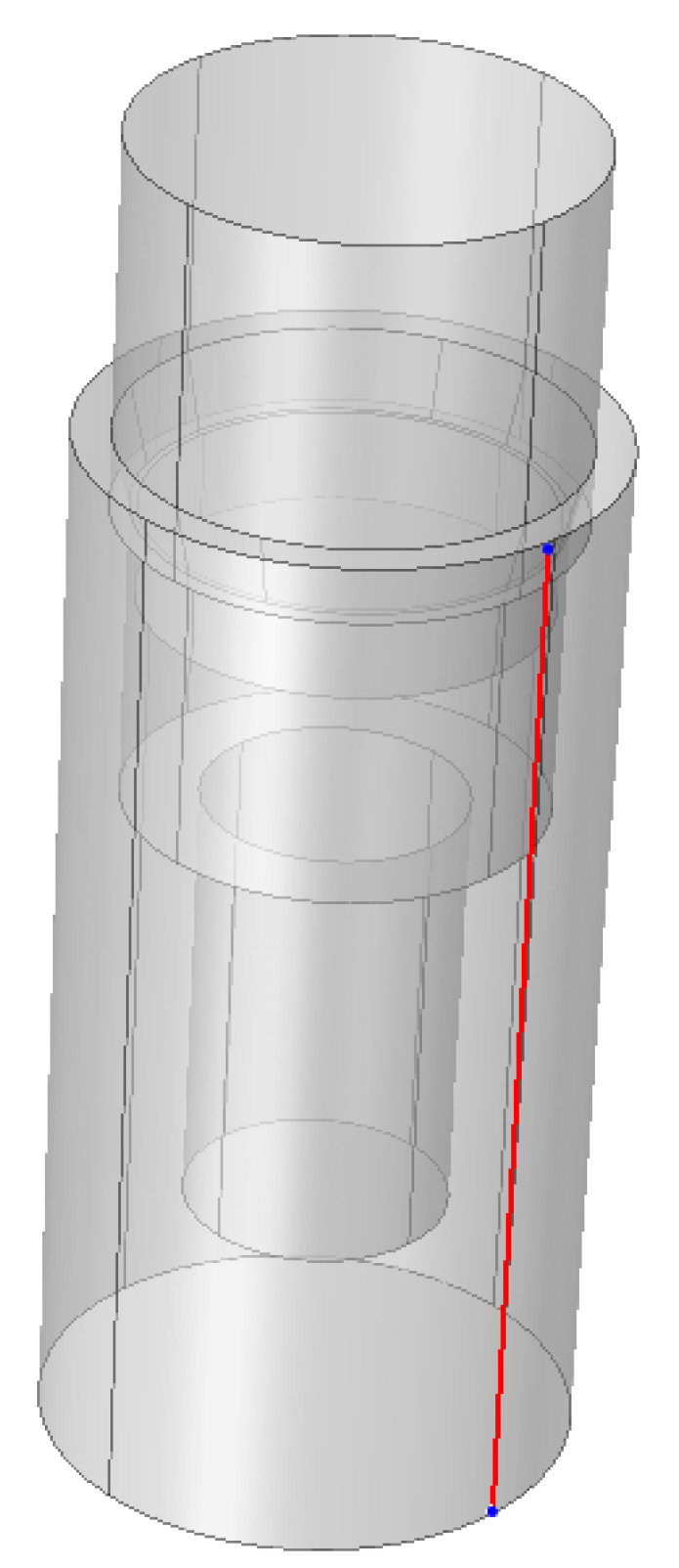
The line defined on the implant side in the case of a 90° connection to assess mechanical stress.

The implants with the smallest (24°) and greatest (90°) cone angle cases available for us were chosen to be included in the FEA. We compressed the models with 400 N at the top. Additionally, a line was defined on the conical surface between the abutment and the implant body (Figure 4) and along the implant height on the side (Figure 5) to evaluate mechanical stress distribution across the defined lines. 

During analyses, Ti material parameters were the following: density: 4500 kg/m^3^, Young’s modulus: 110 GPa, and Poisson’s ratio 0.34. The Yield limit of the CP 4 Ti was considered 480 MPa [39]. Many publications discuss Ti raw material as tough and resistant to plastic deformation [40,41]. 

### 2.5. Statistical Analysis

The results of the measurements were presented as mean ± SEM (standard error of the mean). Statistical analysis was performed using IBM SPSS 23.0 (IBM Corp., Somers, NY, USA) software; one-way analysis of variance (ANOVA) followed by Tukey HSD post-hoc tests were performed on the measured values. During analyses, *p*-values < 0.05 were considered statistically significant.

## 3. Results

### 3.1. Static Load Results

**Figure 6:**
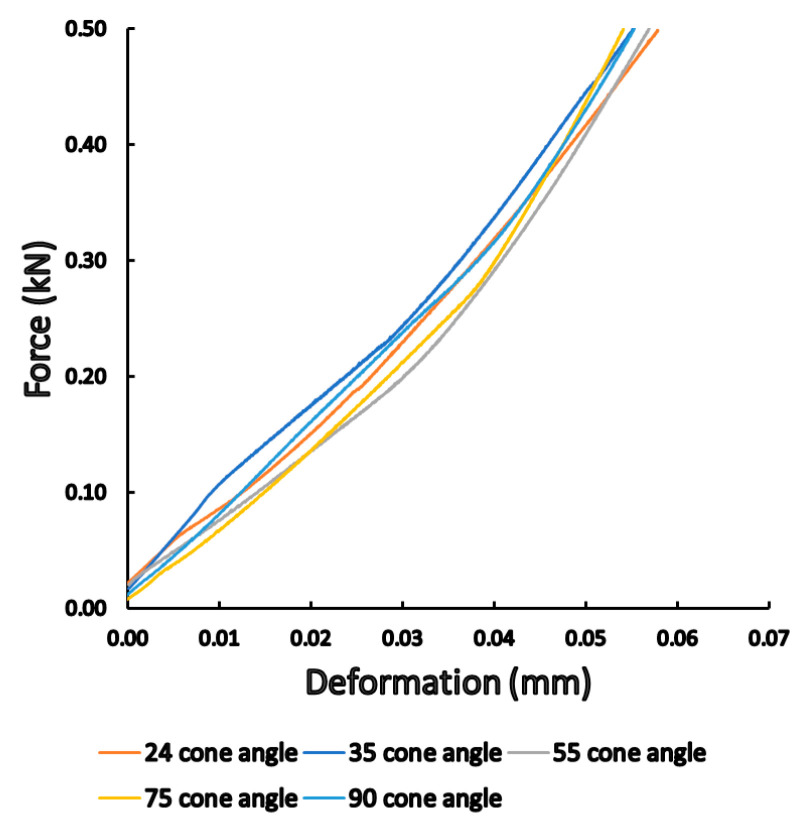
Results of the static load tests. Representative load-compression graphs showed differences among different conical angle implants.

During the static load tests, the device recorded the vertical compression of the abutment into the implant and force. The representative load-compression graphs are presented in Figure 6. For each cone angle (24°, 35°, 55°, 75°, and 90°) group. As the force gradually increased, a linear relationship was observed with the compression, i.e., the load was in the elastic region of Ti. Different conical angle implant–abutment connections showed different load curves, i.e., there were differences in how the compression increased due to the load. The smallest compression was obtained with a cone angle of 75°, while the highest was in the case of 35°.

**Figure 7:**
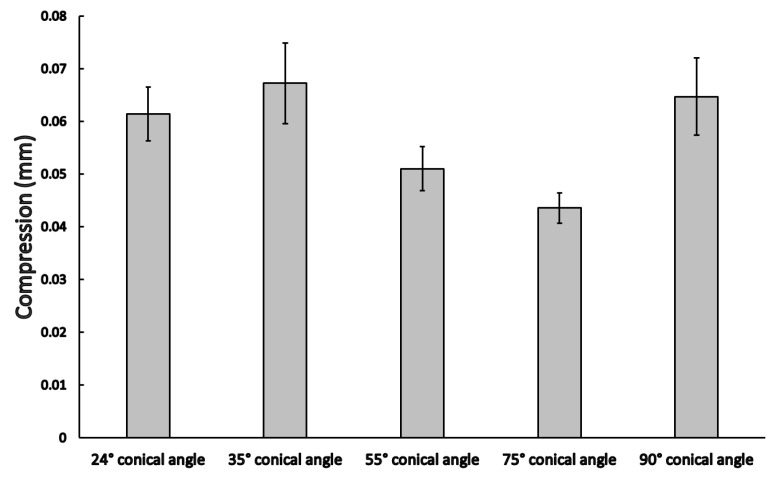
Compression rate (mean ± SEM) among different conical angle implants in the static load tests.

The compression rate of implants with different cone angles was compared at the highest static force value, as shown in Figure 7. Significant differences among the mean compression rates of implants with different cone angles were seen (*p* = 0.021); however, based on post-hoc analyses, only the 35° and 75° cone angle implants were significantly different (0.067 ± 0.008 mm vs. 0.044 ± 0.003 mm; *p* = 0.032).

**Figure 8:**
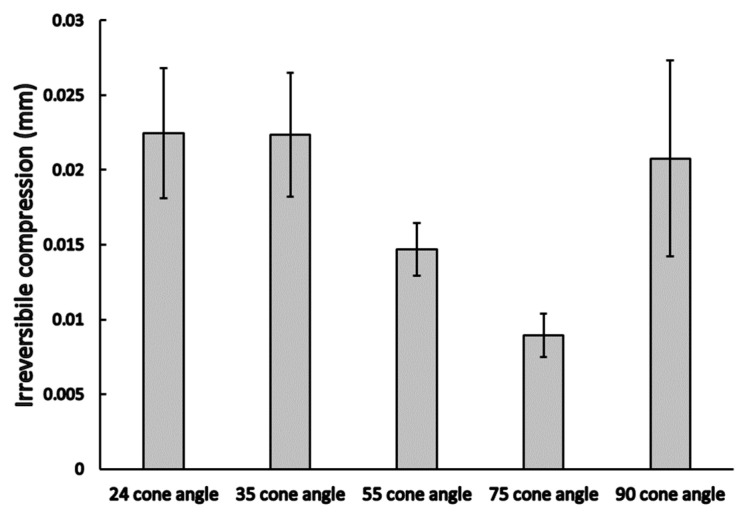
Irreversible vertical compression rate (mean ± SEM) among different conical angle implants and abutment connections.

After the deload, the irreversible vertical compression of the implant and the abutment were determined. Our results showed no significant differences between the different cone angles (*p* = 0.08). Cone angles of 24°, 35°, and 90° showed a similar mean irreversible compression rate of ~0.022 mm (Figure 8); on the other hand, the 75° cone angle case showed the lowest irreversible vertical compression.

**Figure 9:**
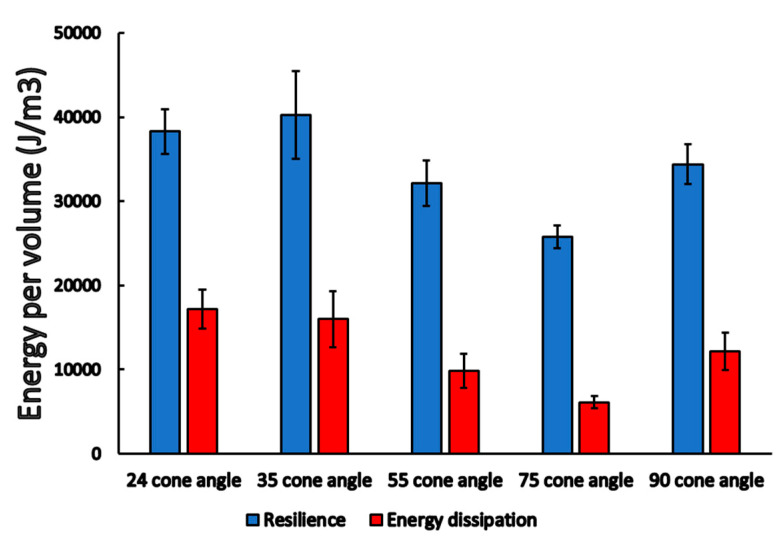
Resilience and energy dissipation (mean ± SEM) among different conical angle implants and abutment connections.

From the area of the load curves, resilience, and energy dissipation were determined. The different cone angle cases showed significant differences both in the case of resilience (*p* = 0.02) and energy dissipation (*p* = 0.01), as demonstrated in Figure 9 and Table 1. We found the highest resilience value in the case of 24° (38,293 ± 2640 $\frac{J}{m^{3}}$), and 35° (40,221 ± 5194 $\frac{J}{m^{3}}$), and, interestingly, the 75° cone angle case showed the lowest resilience value (25,748 ± 1357 $\frac{J}{m^{3}}$). The dissipated energy showed a similar order, 24° (17,165 ± 2325 $\frac{J}{m^{3}}$) and 35° (16,014 ± 3333 $\frac{J}{m^{3}}$) were the highest, and the 75° (6129 ± 731 $\frac{J}{m^{3}}$) case was the lowest.

### 3.2. Dynamic Load Results

**Figure 10:**
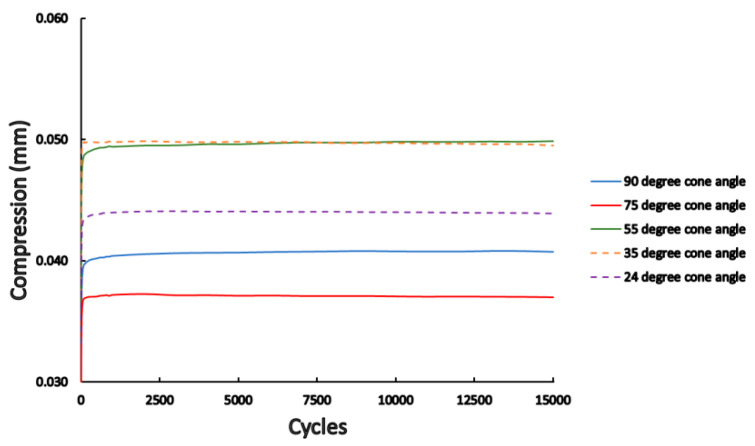
The mean measurements for dynamic compression force among different conical angle implants for all 15,000 cycles.

During the dynamic load test, the fatigue machine recorded the load head position, which was in a direct relationship with the vertical compression of the implants (i.e., how much the abutment slipped into the implant structure). The device also recorded the given force values over time; therefore, load-compression graphs could be analyzed. The load-compression results for the whole 15,000 cycles are presented in Figure 10. It may be observed that there are different degrees of average vertical compression based on the different conical angles. The highest compression was measured in the case of 35° and 55° while the lowest was at the 75° and 90° conical angle implants. Due to the elastic properties during the deload, the material may still deform back. The vertical compression occurred in the very first cycle, while it remains constant thereafter, which may be identified in Figure 10. At the beginning of the compression cycles, there was a sudden rise in compression and after that, there was no change in compression rate. 

**Figure 11:**
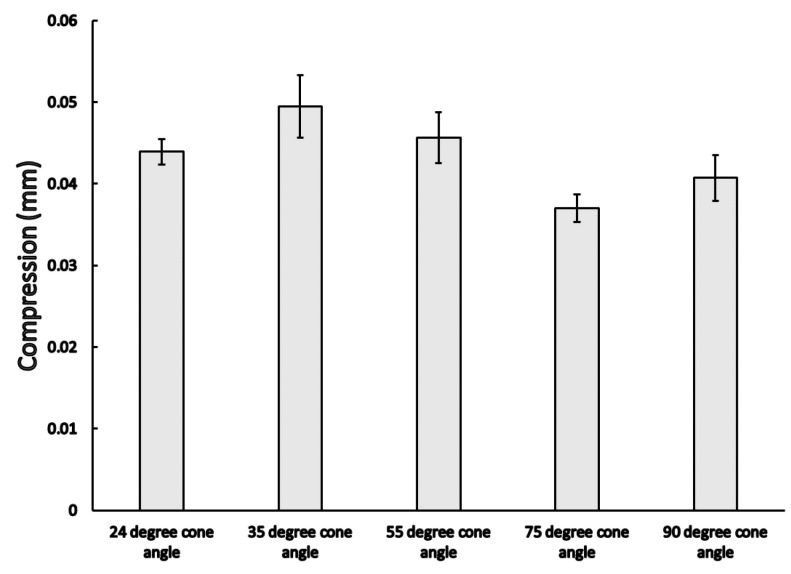
Total vertical compression (mean ± SEM) among different conical angle implants at the 15,000th cycle.

**Figure 12:**
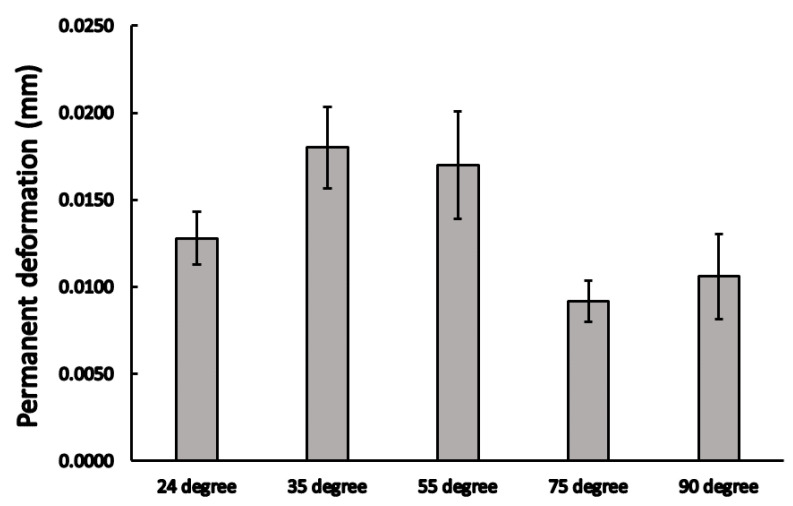
The irreversible (permanent) deformation (mean ± SEM) among different conical angle implants in the implant–abutment system after the dynamic test.

The samples only deformed elastically mainly after this early phase. At the end of the fatigue test, the compression was gathered before the deload phase. The results of the vertical compressions at the end of the dynamic load may be observed from Figure 11, where significant differences were observed among the different cases (*p* = 0.029); comparative analyses showed that there was a significant difference between the 35° and 75° (0.049 ± 0.004 mm vs. 0.037 ± 0.002 mm; *p* = 0.011 see Table 2), and in that case of 55° and 75° conical angle implants (0.046 ± 0.003 mm vs. 0.037 ± 0.002 mm; *p* = 0.009, see Table 2). The permanent deformations were also measured after the dynamic load test: the loading head deloaded the samples and the final position was recorded; these results may be seen in Figure 12, where significant differences between the mean permanent deformations were noted (*p* = 0.032).

**Figure 13:**
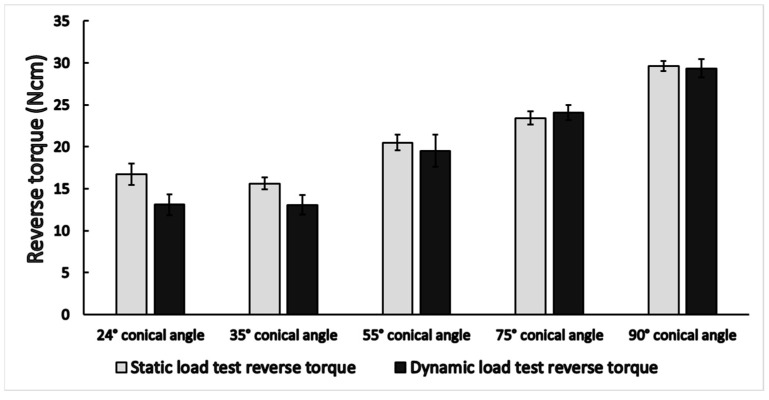
Reverse torque (mean ± SEM) needed to roll apart the implant head and implant after the fatigue test among different conical angle implants.

After the dynamic load test, the lowest torque needed to roll apart the abutment and implant was also measured (Figure 13). The lowest torque values were noted for the 24° case (13.1 ± 1.26 Ncm), while the highest was for the 90° case (29.4 ± 1.1 Ncm). Significant differences were observed between the mean torque both in the case of static and dynamic load tests, (*p* < 0.001 in both cases). With the exception of the 24°- and 35°-degree conical angle connections (*p* = 0.384 and *p* = 0.994), there were significant differences in every other case both after the static and dynamic load test (*p* < 0.05). 

### 3.3. Finite Element Analysis 

**Figure 14:**
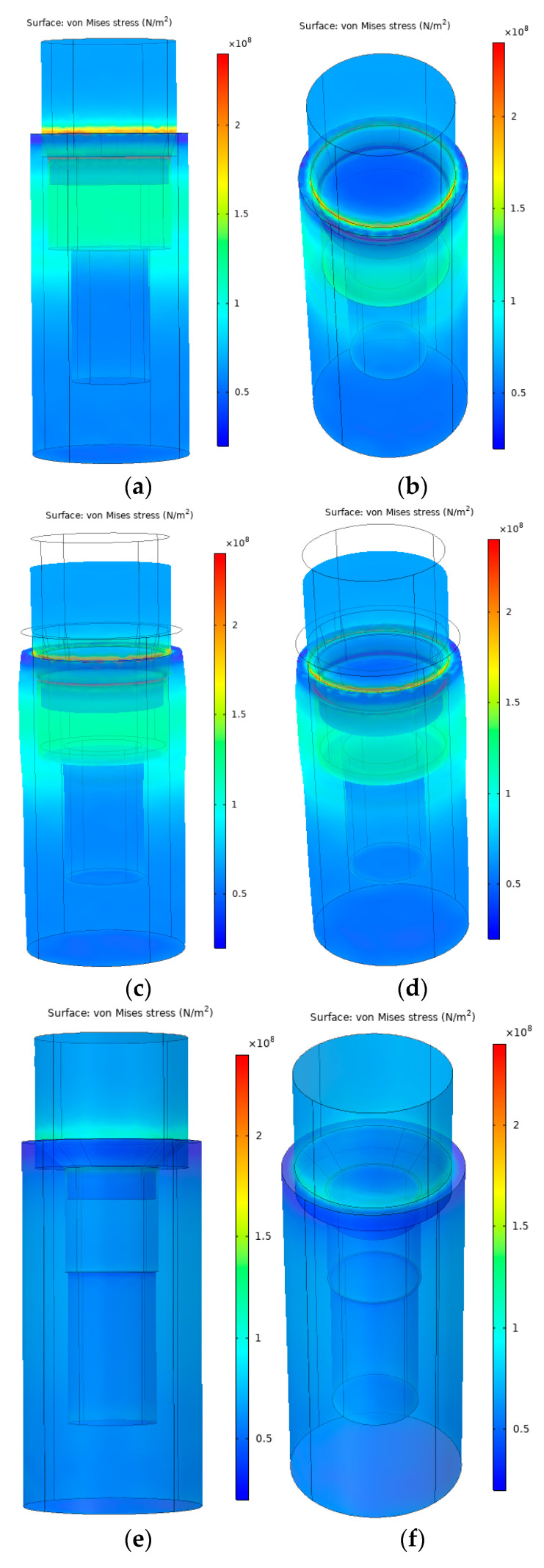

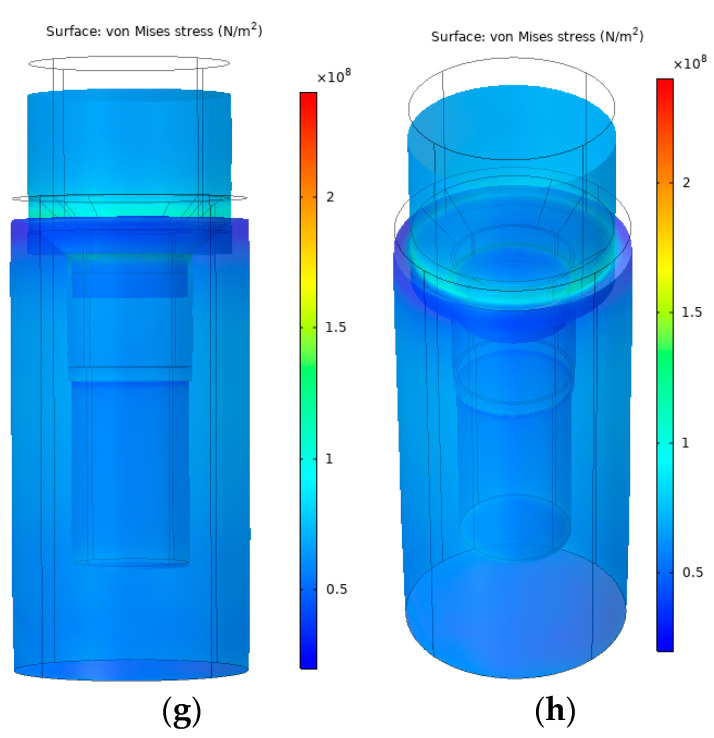
(**a**–**h**). Finite element analyses of the mechanical stresses in case of compression at 24° (**a**–**d**) and 90° (**e**–**h**) conical angle implant and abutment model geometries.

The FEA showed a pronounced difference between the two selected cone angle implant models in von Mises stresses. In the case of 24°, the calculated mechanical stress presenting in the implant was roughly 3 times greater than in the case of 90°. Moreover, around 130 MPa of mechanical stress was concentrated in the upper third of the implant, with the highest stress values seen at the conical surface. Figure 14a,b shows the mechanical stress and deformation in the case of the 24° implant, while Figure 14c,d shows the same mechanical stress but the deformation is scaled by 100. The horizontal deformation, in barrel-shape, can be seen clearly on the scaled Figure 14c. This barrel-shape deformation cannot be observed on the scaled 90° (Figure 14e–h). In the case of a 90° cone angle implant, around 60 MPa mechanical stress was distributed equally on the implant wall and the mechanical stress peaked at the conical connection. The highest mechanical stress value noted was around 300 MPa in the case of 24°, while it was only around 160 MPa in the case of the 90° conical angle case.

**Figure 15:**
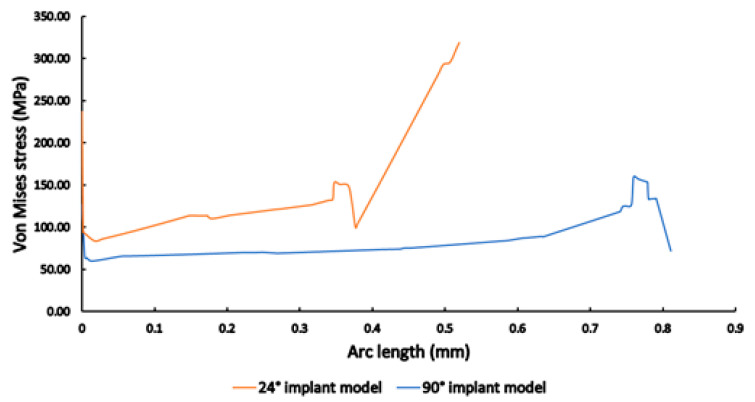
The mechanical stress distribution along the selected line on the abutment and implant connection in the case of 24° (orange) and 90° (blue) conical angles.

**Figure 16:**
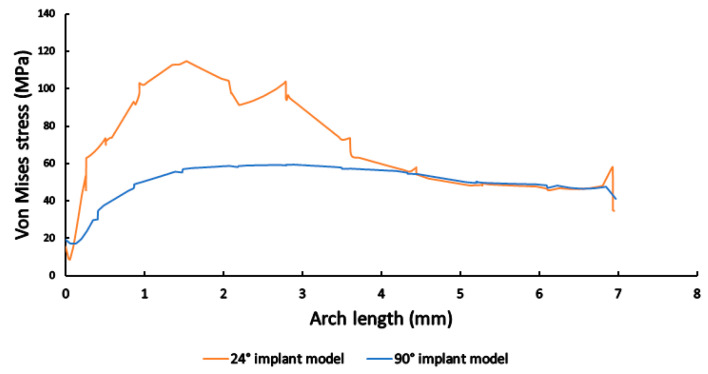
The mechanical stress distribution along the implant height on the side in case of 24° (orange) and 90° (blue) conical angles.

The results of mechanical stress distribution measurements along the selected line on the abutment and implant connection and along the implant height on the side are shown in Figure 15 and Figure 16, respectively. In both cases, it may be clearly observed that stresses were much higher in the case of the 24° implant model; on the abutment and implant connection, stress values were consistently higher (>100 MPa, compared to the 90° implant model with values ~60–70 MPa), with a sharp run-off to higher values (Figure 15); while along the implant height on the side, the 24° implant model reached stress values near 120 MPa, while stresses were consistently near 60 MPa in the case of the 90° model (Figure 16).

## 4. Discussion

The aim of our study was to understand the effect of different taper-angle implant abutment relationships on the long-term survival and clinical success of dental implants. During our investigation, two issues were identified: on the one hand—according to the results of the mechanical tests—the screw loosening, as well as the horizontal deformation, based on the results of the FEA. As the cone angle of the implant superstructure connection increased, the extraction torque of our fixing screw decreased proportionally, i.e., the larger the connection angle, the loosening of the screw under load was smaller. The management of screw loosening is a common issue in clinical practice; the change in the torque is affected by the continuous, periodical repeating loads. Our static and dynamic results both showed the highest change in the case of lower conical angles. This resulted in the decrease of the reverse torque needed to take apart the implant and the abutment. However, increasing the conical angle of the abutment improved the results, i.e., it has led to lower rates of compression and less decrease in the case of the small angle cases. 

Irreversible vertical deformation may also cause compression of the implant and the abutment, if the taper angle of the connection is small, a phenomenon that may be exacerbated by manufacturing inaccuracy, i.e., the height of the implant and superstructure may change as a result of the load. In this case, the occlusal height also decreases, thereby changing the occlusion, which leads to further biological (malocclusion, traumatic occlusal forces, peri-implant bone loss, temporomandibular dysfunction) and mechanical (screw loosening, fracture, superstructure deformation, fracture) issues [42]. The inaccuracy of the taper angles of the implant and the abutment has a significant influence on the compression under load. The greater the dimensional error from manufacturing, the greater the conical surface shrinkage [43]. Ti is traditionally described as a material that is tough and resistant to plastic deformation; the resistance of Ti to fatigue may be measured by the rotating cantilever beam test, during which the strains are predominately elastic, both upon initial loading and throughout the test, in accordance with the ASTM E466. Grade 4 Ti material has a fatigue limit ~310 MPa [44]. During our experiments, we did not experience the plastic properties of Ti during fatigue, in addition, there was no force that would exceed the yield strength of Ti, according to our FEA results. However, it has been described by Zhao et al. that low-velocity impact damages could decrease the compressive failure strength of Ti honeycomb sandwich structures by up to 15% [45]. While this was not studied in our tests, fatigue due to cyclic loading may cause changes in the material yield stresses and stress concentrations, in which case the plastic property of the Ti alloy theoretically could have had an impact on the results. 

Of note, the widely accepted view that inadequate occlusion may lead to biological complications is poorly reported in the literature, and it is difficult to demonstrate that the consequences are pathognomonic for the presence of overload [46]. Even with the most modern digital technologies, the applied tests cannot give a reproducible, quantifiable absolute or relative value [47]. In clinical conditions, solo or bridge stuttering may have different consequences. This can also cause rotation in the case of single dentures or single dentures with a cantilever. After the static load test, we also performed a high load measurement in each group, where the implant abutment contact was loaded with a 2 kN force. The result was that there was no measurable extension torque in the fixing screw because the degree of compression was greater than the thread height of the fixing screw. Because of this, the connection of the implant to the abutment was not damaged, and the implant did not break.

Based on the FEA methodology and models, many studies showed that implant geometry, bone quality, and site of implant placement affect load transmission mechanisms, thereby, subsequently, also affecting peri-implant bone resorption [48]. Maximum stress areas may be located at the implant neck, and possible overloading could occur in the form of compression in the compact bone (due to lateral components of the occlusal load) and in the form of tension at the interface between the cortical and trabecular bone (due to vertical intrusive loading components) [48,49,50]. Load transmission may also occur in the abutment–implant interface zone, which may also lead to the above-mentioned phenomenon [51,52,53]. 

Our FEA also confirmed that the implant–abutment connection greatly influences the distribution of forces in different ways at different heights between the implant and the bone. In the FEA, the mechanical stress was better distributed over the entire surface of the implant in the case of the 90-degree implant–abutment connection, compared to the 24-degree connection. Regarding the 24-degree connection, the mechanical stress was greatest in the area where the cones meet, which represents the part of the implant with the smallest wall thickness; in addition, in the case of the 24-degree model, not only vertical but also horizontal deformation occurs. This horizontal deformation may lead to peri-implant bone resorption in the cortical bone. When the taper angle is increased, more of the load is transferred to the implant wall than to the fixing screw. For this reason, the higher load on the smaller taper angle resulted in greater screw loosening, as more force is transmitted to the screw.

In the study of Paepoemsin et al., the removal torque of three different types of abutment screws was evaluated after mechanical cyclic loading. In their paper, flat head and tapered screws were used, and after the first 10 min of dynamic load and after 1 million cycles, they compared the reverse torques of each group. Similarly, to our results, they have shown a statistically significant decrease in the measured reverse torque. Our results also indicated that during the dynamic load test, there was a change, which decreased the tightness of the grip of the abutment head into the implant [54]. Benjaboonyazit et al. also studied the emergence of loose connections due to fatigue. In their experiments, they used 3.75 mm Octatorx-cone implants and tightened the screws with 30 Ncm force, after which, a very long, 2 million cycle dynamic load test was performed. Their results were also consistent with our findings, i.e., without any load, they obtained reverse torque values over 27 Ncm, which decreased to less than 16 Ncm after their fatigue test protocol. However, our study showed that this decrease in the reverse torque may be moderated with an increased conical angle. Comparing the static 55° results, which resulted in 24–25 Ncm reverse torque, after the dynamic load test, the 60° case decreased to very similar values, i.e., around 24 Ncm. These results also highlighted that increasing the conical angle indeed helps maintain a stronger grip for longer periods even in the case of periodical loads [55]. Joo-Hee and Hyun-Suk performed cyclic load tests as well on Grade 4 Ti implants with an external hex connection. To follow the International Organization for Standardization (ISO) protocol, they loaded the implants at a 30° angle, and after a 1 million cycle of 300 N (which is equivalent to 30 kg) force, they measured the reverse torque at 15.2 Ncm; they also determined the torque values before the dynamic test, which was 25.2 Ncm. Their results coincide with the findings of the present study, as, in their case, the decrease in the reverse torque was roughly 40% compared to the pre- to post-dynamic test [56]. The above-referenced articles support the results of our tests, i.e., under loading, the greater the angle of the implant–superstructure connection was, the smaller the amount of screw loosening could be measured.

The formation of biological width after implant placement is an important factor in the prevention of peri-implant bone loss [57]. Adaptation and remodeling of these soft tissues may have considerable roles in facilitating secondary stability and the long-term survival of implants, as they absorb forces that act on the implant, thus reducing the transmission of forces acting on the jawbone. The viscoelastic absorbent properties of soft tissues around the implant have been described, both at the cellular [58] and tissue levels [59], respectively. The forces acting on the dental prosthesis are distributed and continue to affect the superstructure, the implant, the implant connection, and the bone. Through the implant–abutment connection, the mentioned forces may cause loosening of the fixing screw, irreversible vertical compression, and overloading of the bone in different ways at different heights depending on the design. Manufacturing inaccuracies in the implant–abutment connection may cause both mechanical and biological problems.

In order to achieve long-term implantation success—from a mechanical point of view—it may be crucial that the prosthetic phase takes place at the implant or abutment levels. Important examples from clinical practice may include situations with a large axis deviation, where the contact part of the abutment is reduced in the dental technique phase in order to facilitate the placement of the restoration. However, this may have a detrimental effect on the fit between the implant and the abutment, thereby changing the distribution of masticatory forces between the implant body and the surrounding bone; in many cases, this may lead to the breakage of the screw securing the restoration or the fracture of the bridge itself.

The implant–abutment relationship affects the indications in which the implants may be used. It is extremely important that in the case of special indications (immediate implantation, immediate loading, or cracking technique) the implant–abutment connection should serve the best possible force distribution on the surface of the implant, i.e., transfers the masticatory force to the bone on the largest possible surface.

## 5. Conclusions

According to our mechanical tests, the amount of screw loosening changes significantly with the change in the taper angle of the connection. The larger the angle of the implant–superstructure connection, the smaller the screw loosening due to loading. As a result of our finite element analyses, it can be concluded that, in the case of connections with a smaller taper angle, the masticatory forces acting on the implants were concentrated on the upper third of the implant body; this may result in horizontal deformation in the implant neck, which could lead to increased risk of cortical bone resorption. While in the case of a larger taper angle, the masticatory force is evenly distributed over the body of the implant, this is also determined by a number of other factors, including the thread design, shape, length, and diameter of the implant. Our results may contribute to the understanding of the long-term success of dental implants.

## Figures and Tables

**Table 1:** Results of the post-hoc tests (*p*-values) during pairwise comparisons of resilience (blue) and energy dissipation (red) among different conical angle implants. *p*-values < 0.05 are presented in **boldface**.

	24°	35°	55°	75°	90°
24°	-	0.721	**0.030**	**0.002**	0.145
35°	0.666	-	0.065	**0.004**	0.260
55°	0.174	0.077	-	0.250	0.493
75°	**0.008**	**0.003**	0.159	-	0.079
90°	0.404	0.215	0.626	0.070	-

**Table 2:** Results of the post-hoc tests (*p*-values) during pairwise comparisons of mean vertical compressions among different conical angle implants. *p*-values < 0.05 are presented in **boldface**.

	24°	35°	55°	75°	90°
24°	-				
35°	0.234	-			
55°	0.204	0.932	-		
75°	0.141	**0.011**	**0.009**	-	
90°	0.490	0.065	0.055	0.422	-

## Data Availability

All data generated during the study are presented in this paper.
